
JOURNAL INFORMATION
==============================
NLM Title Abbreviation: ScientificWorldJournal
Journal ID: ScientificWorldJournal
NLM Journal ID: 101131163
Journal ID: TSWJ

The Scientific World Journal

ISSN: 2356-6140
EISSN: 1537-744X
Publisher: Wiley

ARTICLE INFORMATION
==============================
PMCID: PMC5763774
PMID: 20495770
DOI: 10.1100/tsw.2010.88
Article ID: 507693
Article version: 1
Subjects: Directions in Science

Sepsis-Induced Degradation of Endothelial Glycocalix

Henrich Michael *
Gruss Marco
Weigand Markus A.
Department of Anesthesiology, Intensive Care Medicine and Pain Therapy, Justus-Liebig-University Giessen, Germany
*Michael Henrich: michael.henrich@anatomie.med.uni-giessen.deAcademic Editor: John P. Williams

Publication date: 2010
Electronic publication date: 2010 May 18
Volume: 10
First page: 917
Last page: 923
Received 2010 Feb 5; Revised 2010 Apr 18; Accepted 2010 Apr 19
Copyright: Copyright © 2010 Michael Henrich et al.
Copyright year: 2010
License: This is an open access article distributed under the Creative Commons Attribution License, which permits unrestricted use, distribution, and reproduction in any medium, provided the original work is properly cited.
License URL: https://creativecommons.org/licenses/by/3.0/

Keywords: sepsis, endothelium, glycocalix, proteoglycans, glycoproteins, glycosaminoglycans, syndecan-1, heparan sulfate, abdominal surgery

==============================
Directions in Science
*TheScientificWorldJOURNAL* (2010) 10, 917–923
ISSN 1537-744X; DOI 10.1100/tsw.2010.88

TheScientificWorldJOURNAL
www.thescientificworld.com

### Sepsis-Induced Degradation of Endothelial Glycocalix

Michael Henrich*, Marco Gruss, and Markus A. Weigand

*Department of Anesthesiology, Intensive Care Medicine and Pain Therapy, Justus-Liebig-University Giessen, Germany*

**E-mail:** michael.henrich@anatomie.med.uni-giessen.de; marco.gruss@chiru.med.uni-giessen.de; markus.weigand@chiru.med.uni-giessen.de

*Received February 5, 2010; Revised April 18, 2010; Accepted April 19, 2010; Published May 18, 2010*

---

**KEYWORDS:** sepsis, endothelium, glycocalix, proteoglycans, glycoproteins, glycosaminoglycans, syndecan-1, heparan sulfate, abdominal surgery

---

### INTRODUCTION

Sepsis, a systemic host response to microbial infections, is a major cause of death in intensive care units, with mortality rates up to 60%[1,2]. A major pathophysiological process in sepsis is dysregulation of circulation, mainly caused by altered vasomotion, redistribution of organ blood flow, impaired rheology and deformability of red and white blood cells, occlusion of vessels, and loss of endothelial barrier function[3]. This directly induces reduced tissue oxygen supply and causes organ failure[4]. Within this process, the arterial endothelium plays a pivotal role, regulating the vessel resistance and fluid homeostasis between blood and the interstitial space under physiological conditions[5]. Fluid and solute balances between vessels and tissue, either transcellular or paracellular, underlie a fine regulation of the endothelium[6,7]. The active transcellular transport of molecules contributes only little to the vast fluid extravasation during inflammation. It is mainly the passive paracellular transport that is responsible for the major exchange between vessels and tissue, which is also controlled by precise regulation mechanisms[8]. A key function in regulation of these processes is played by the glycocalix. This fine structure decorates the luminal membrane of endothelial cells and was first identified 40 years ago while utilizing special electron microscopic staining techniques[9]. The side branches of the glycocalix consist of negatively charged proteoglycans, glycoproteins, and glycosaminoglycans[10,11,12]. The three core proteins of the proteoglycans found on endothelial cells are the transmembrane syndecan, the basement matrix-associated perlecans, and the membrane-bound glypicans[13,14]. The glycocalix extends luminally from several nanometers to 3 µm, and its dimension is determined by the dynamic balance between biosynthesis, conditions of its microenvironment, such as cation content and concentration, pH, and shear-dependent or enzymatic shedding of its constituent parts[15,16,17,18,19,20,21]. This fragile luminal mesh is responsible for a constant fluid resistance and it interacts with plasma proteins. While binding with its negatively charged heparan sulfate proteoglycans to antithrombin III, it constrains factor Xa-activity and inhibits coagulation[22,23]. It is assumed that the fine structure of the glycocalix is a mechanotransducer that enables the endothelial cells to sense changes in blood flow. In particular, increased shear stress evokes modulation of the vasomotion by the generation of nitric oxide[12,24,25,26]. Furthermore, the three-dimensional glycocalix contributes to the endothelial sieve

*Corresponding author.
©2010 with author.
Published by TheScientificWorld; www.thescientificworld.com function that is mainly determined by the hyaluronic acid and chondroitin side chains[27,28,29,30]. These negatively charged side chains restrain plasma components as fluid and proteins in the vessel lumen, thus preventing interstitial edema[5,31,32]. Furthermore, the glycocalix is linked to the cortical cytoskeleton of endothelial cells and acts as a transducer for mechanical and chemical stimuli[33,34]. Via this pathway, it influences the activation of cytoskeletal actin filaments, which are linked to the intercellular junction proteins cadherins and integrins[35]. As a response to stimuli onto the glycocalix, it induces retraction and reorganization of the cytosolic actin filaments and of their attached intercellular junctions, hence enhancing endothelium permeability[26,35].

### SEPSIS- AND SURGERY-INDUCED DAMAGE OF GLYCOCALIX

A recent study published in the *Journal of Surgical Research* investigated the deterioration of the vascular glycocalix in patients with sepsis or after major abdominal surgery, and compared these findings with healthy volunteers. Within this clinical study, it has been demonstrated that in patients either with severe sepsis or after major abdominal surgery, the plasma concentrations of glycocalix markers (syndecan-1, heparan sulfate) increased by the same extent. This implies that under both situations, the glycocalix is shed off the endothelium. In sepsis, the glycocalix marker syndecan-1 correlates with concentrations of inflammatory markers (intercellular adhesion molecule-1 [ICAM-1], vascular cell adhesion molecule-1 [VCAM-1], and mainly with interleukin-6 [IL-6]), which were significantly higher than after abdominal surgery[36]. During sepsis and after abdominal surgery, the plasma leaks out into the interstitial space, and this has great impact on the development of edema and impaired oxygen and nutrient supply to tissues. The deterioration of the endothelial glycocalix is one of the earliest steps within this scenario that triggers the loss of endothelial barrier function[11].

### VASCULAR PERMEABILITY

The importance of the glycocalix has been underestimated for a long time, however, during the past years, it became apparent that this structure plays a major role in regulating circulatory fluid homeostasis and vessel integrity[4,5]. During sepsis and abdominal surgery, the glycocalix is disintegrated and its components can be quantified within the plasma, reflecting peripheral endothelial cell injury[36]. However, an earlier study provided evidence that in patients with septic shock, the plasma concentrations of glycosaminoglycans increase and are highest in nonsurvivors compared to survivors[37]. Consequently, the confirmation of glycocalix components within the plasma can be used to diagnose the severity of septic processes, because they provide evidence of endothelial barrier damage and impairment of microcirculation. Within the plasma, the separated heparan sulfate compounds contribute to the recruitment of leukocytes, acting as a chemotactical positive feedback mechanism by leading leukocytes to the site of inflammation[38,39]. Beside sepsis and surgical trauma, the glycocalix is also degraded during ischemia/reperfusion after vascular surgery, and hyperglycemia leads to damage of the glycocalix in diabetes type 1. All these pathophysiological processes are accompanied by increased vascular permeability, by opening paracellular pathways and the interruption of intercellular junctions. This impairment of the endothelial barrier can be evoked by inflammatory mediators such as tumor necrosis factor alpha (TNF-α), bradykinin, thrombin, vascular endothelial growth factor (VEGF), and histamine acting directly on endothelial cells[40,41,42,43]. Increased paracellular permeability is also induced by damage of the glycocalix during inflammation, since this structure contributes to the fluid balance in tissues[44,45,46,47]. Additionally, it has been shown that the integrity of the glycocalix is impaired by TNF-α or lipopolysaccharide (LPS); both inflammatory mediators reduce the thickness of the glycocalix, hence, extravasation of fluid and solutes are triggered[28,48,49]. During inflammation, the glycocalix also acts as a receptor for cytokines that leads to a rearrangement of syndecans to clusters within the luminal endothelial cell membrane[50]. Mainly, the syndecans integrate extracellular signals by their association to cytosolic effectors, such as the actin filaments[51]. In a following step, actin filaments change their order to stress fiber formation and intercellular junctions become more permeable, allowing fluid extravasation[50]. Consequently, the glycocalix contributes to the modulation of endothelial barrier function during (patho-)physiological conditions via different pathways.

### COMPARISON OF SEPSIS- AND SURGERY-INDUCED GLYCOCALIX DEGRADATION

The study of Steppan et al.[36] compared changes in the glycocalix in patients suffering from sepsis with patients after abdominal surgery and with healthy individuals. The aim was to provide evidence that sepsis and abdominal surgery cause significant flaking of the endothelial glycocalix. The study compared a total of 150 individuals, including 104 patients with severe sepsis, 28 patients after abdominal surgery, and 18 healthy volunteers. In the sepsis group, the 28-day mortality after diagnosis was 47%; the initial site of infection was either the lung (58.7%), genitourinary tract (11.6%), gastrointestinal tract (3.8%), surgical site (6.7%), or other (10.6%). Blood samples were either taken at the time point of diagnosis, or briefly after surgery, 6, 24, and 48 h later, whereas blood samples from healthy volunteers were taken once. The plasma concentrations of glycocalix components, such heparan sulfate, syndecan-1, IL-6, ICAM-1, and VCAM-1, were quantified by applying enzyme-linked immunosorbent assays (ELISA). The study found significantly higher concentrations of IL-6, ICAM-1, and VCAM-1 in patients with severe sepsis than in surgical patients or in healthy people. In surgical patients, the inflammatory marker IL-6 was significantly increased compared to healthy individuals, whereas ICAM-1 and VCAM-1 showed no difference between both groups. In the sepsis and surgical groups, the concentrations of glycocalix markers syndecan-1 and heparin sulfate were significantly higher than in the control group. In surgical patients, the heparan sulfate levels were higher than in the sepsis group; on the other hand, syndecan-1 was more elevated in the sepsis group than in the surgical group. There was no difference of these two markers between patients who survived or patients who died during sepsis. The levels of IL-6 correlated with levels of VCAM-1, ICAM-1, syndecan-1, and lactate. Both sepsis and abdominal surgery cause flaking of the glycocalix, which is in line with findings that both cause vascular leaking. However, the damage of the glycocalix in sepsis is more substantial, which is reflected by the increased levels of the cell membrane molecule syndecan-1 levels. However, the syndecan residues that are shed function as important signal molecules during the subsequent inflammation cascade. In murine sepsis studies, syndecan-1 shedding is associated with removal of tissue-bound CXC chemokines and this further facilitates resolution of neutrophilic inflammation[52]. So syndecan-1 may play a pivotal role during sepsis by regulating the host response. This is supported by findings in mice where thermal injuries cleave syndecan-1 and this allows the spreading of *Pseudomonas aeruginosa* assumed by enhanced vascular permeability. Furthermore, syndecan-1 null mice are less susceptible to systemic *Pseudomonas* infections. They show lower mortality rates and significantly lower levels of proinflammatory cytokines[53]. On the contrary, syndecan-1 shedding protects from Gram-positive toxic shock; it inhibits amplification and dysregulation of the host inflammatory response[54]. So shedding of the glycocalix contributes to increased vascular permeability, by a reduced sieve function, and by rearranging cytoskeletal proteins and altering intercellular junctions.

### CLINICAL ASPECTS

The degradation of the glycocalix during sepsis and major abdominal surgery has a great impact on fluid balance and the development of edema. However, so far, there exist no methods to evaluate endothelial dysfunction and vascular leakage during sepsis and during the postoperative period; therefore, diagnosis of fluid distribution during these scenarios is very difficult[55]. The findings of Steppan et al.[36] that the glycocalix is damaged during major abdominal surgery and sepsis can explain the large fluid shifts into the interstitial space. It has been estimated that during major surgery, a fluid shift of 3–6 l occurs; beyond that in septic patients, an extracellular overload of 10 l has been demonstrated after 2 days of treatment[56,57,58,59,60]. This overinfusion ought to be avoided, since it further worsens oxygen supply of tissues and organs[4]. Still, there exists no routine bedside method to quantify interstitial edema, blood volume, or extracellular fluid compartments. Anyway, it is important to maintain hemodynamic stability during sepsis and after abdominal surgery. Currently, the hydration conditions have to be appropriately maintained by sufficient fluid supply. Applied crystalloid solutions, omitting any osmotic forces, do not remain intraluminally even in the presence of an intact glycocalix and physiological vascular permeability. These solutions are distributed over the entire extracellular space, which consists of interstitial (80%) and intravascular (20%) compartments[31,32]. This provides reason for the observation that administered crystalloid boluses stabilize only insufficiently impaired perioperative hemodynamics[31]. To achieve stable hemodynamics in sepsis, the administration of 4–6 l of crystalloid solutions or 1.5–3 l of colloidal solutions are required[61]. Colloidal solutions remain almost completely intravascular and can enhance cardiac preload in situations with an intact endothelial barrier[31]. However, during impaired endothelial barrier function, colloidal solutions also distribute into the interstitial space and can aggravate edema[62]. Furthermore, colloidal solutions, such as pentastarch, with higher substitution grades can provoke kidney injury and impair patient outcome in sepsis[63]. To avoid iatrogenic overinfusion, the fluid administration should be adjusted to specific hemodynamic parameters, as it has been suggested in clinical studies to achieve adequate circulatory functions[1,64]. The fluid therapy should not be monitored by heart rate, blood pressure, or cardiac filling pressures, such as central venous pressure or pulmonary capillary wedge pressure, only. Instead, dynamic parameters, such as stroke volume, cardiac index, capillary perfusion, and urine production, should be taken into account for monitoring fluid therapy[1,65,66,67]. Nevertheless, the endothelial barrier is deteriorated during sepsis and major abdominal surgery, but colloidal solutions can rapidly stabilize cardiocirculatory dynamics. This should be a fluid-saving therapy without impairing further edema, tissue oxygenation, and endothelial function. Therefore, monitoring glycocalix compounds in the bloodstream may be employed for longer periods in order to determine further endothelial barrier damage or amelioration of endothelial function.

### CONCLUSION

The glycocalix is shed during inflammation and nonspecifically after surgery, explaining capillary leaking. Future studies should focus on how the glycocalix is degraded in sepsis and after abdominal surgery. This understanding will be pivotal in order to develop therapeutic strategies to preserve the glycocalix and, consequently, this will possibly reduce vascular leaking, with its fateful impact on organ functions, and improve the outcome of patients after abdominal surgery and sepsis.

### REFERENCES

1. Dellinger, R.P., Levy, M.M., Carlet, J.M., Bion, J., Parker, M.M., Jaeschke, R., Reinhart, K., Angus, D.C., Brun-Buisson, C., Beale, R., et al. (2008) Surviving Sepsis Campaign: international guidelines for management of severe sepsis and septic shock: 2008. *Intensive Care Med.* **34,** 17–60.
2. Friedman, G., Silva, E., and Vincent, J.L. (1998) Has the mortality of septic shock changed with time? *Crit. Care Med*. **26,** 2078–2086.
3. Spronk, P.E., Zandstra, D.F., and Ince, C. (2004) Bench-to-bedside review: sepsis is a disease of the microcirculation. *Crit. Care* **8,** 462–468.
4. Chappell, D., Westphal, M., and Jacob, M. (2009) The impact of the glycocalyx on microcirculatory oxygen distribution in critical illness. *Curr. Opin. Anaesthesiol*. **22,** 155–162.
5. Pries, A.R., Secomb, T.W., and Gaehtgens, P. (2000) The endothelial surface layer. *Pflugers Arch*. **440,** 653–666.
6. Milici, A.J., Watrous, N.E., Stukenbrok, H., and Palade, G.E. (1987) Transcytosis of albumin in capillary endothelium. *J. Cell Biol*. **105,** 2603–2612.

7. Predescu, S.A., Predescu, D.N., and Malik, A.B. (2007) Molecular determinants of endothelial transcytosis and their role in endothelial permeability. *Am. J. Physiol. Lung Cell. Mol. Physiol.* **293,** L823–842.
8. Michel, C.C. and Curry, F.E. (1999) Microvascular permeability. *Physiol. Rev*. **79,** 703–761.
9. Luft, J.H. (1966) Fine structures of capillary and endocapillary layer as revealed by ruthenium red. *Fed. Proc*. **25,** 1773–1783.
10. Jackson, R.L., Busch, S.J., and Cardin, A.D. (1991) Glycosaminoglycans: molecular properties, protein interactions, and role in physiological processes. *Physiol. Rev*. **71,** 481–539.
11. Noble, M.I., Drake-Holland, A.J., and Vink, H. (2008) Hypothesis: arterial glycocalyx dysfunction is the first step in the atherothrombotic process. *QJM* **101,** 513–518.
12. Reitsma, S., Slaaf, D.W., Vink, H., van Zandvoort, M.A., and oude Egbrink, M.G. (2007) The endothelial glycocalyx: composition, functions, and visualization. *Pflugers Arch*. **454,** 345–359.
13. Rosenberg, R.D., Shworak, N.W., Liu, J., Schwartz, J.J., and Zhang, L. (1997) Heparan sulfate proteoglycans of the cardiovascular system. Specific structures emerge but how is synthesis regulated? *J. Clin. Invest*. **99,** 2062–2070.
14. Weinbaum, S., Tarbell, J.M., and Damiano, E.R. (2007) The structure and function of the endothelial glycocalyx layer. *Annu. Rev. Biomed. Eng*. **9,** 121–167.
15. Coombe, D.R. and Kett, W.C. (2005) Heparan sulfate-protein interactions: therapeutic potential through structure-function insights. *Cell. Mol. Life Sci.* **62,** 410–424.
16. Hileman, R.E., Fromm, J.R., Weiler, J.M., and Linhardt, R.J. (1998) Glycosaminoglycan-protein interactions: definition of consensus sites in glycosaminoglycan binding proteins. *Bioessays* **20,** 156–167.
17. Kan, M., Wang, F., To, B., Gabriel, J.L., and McKeehan, W.L. (1996) Divalent cations and heparin/heparan sulfate cooperate to control assembly and activity of the fibroblast growth factor receptor complex. *J. Biol. Chem*. **271,** 26143–26148.
18. Lipowsky, H.H. (2005) Microvascular rheology and hemodynamics. *Microcirculation* **12,** 5–15.
19. McGee, M.P. and Liang, J. (2001) Regulation of glycosaminoglycan function by osmotic potentials. Measurement of water transfer during antithrombin activation by heparin. *J. Biol. Chem.* **276,** 49275–49282.
20. van den Berg, B.M., Vink, H., and Spaan, J.A. (2003) The endothelial glycocalyx protects against myocardial edema. *Circ. Res*. **92**, 592–594.
21. van Haaren, P.M., VanBavel, E., Vink, H., and Spaan, J.A. (2003) Localization of the permeability barrier to solutes in isolated arteries by confocal microscopy. *Am. J. Physiol. Heart Circ. Physiol.* **285,** H2848–2856.
22. Jacob, M., Rehm, M., Loetsch, M., Paul, J.O., Bruegger, D., Welsch, U., Conzen, P., and Becker, B.F. (2007) The endothelial glycocalyx prefers albumin for evoking shear stress-induced, nitric oxide-mediated coronary dilatation. *J. Vasc. Res*. **44,** 435–443.
23. Kelly, R., Ruane-O'Hora, T., Noble, M.I., Drake-Holland, A.J., and Snow, H.M. (2006) Differential inhibition by hyperglycaemia of shear stress- but not acetylcholine-mediated dilatation in the iliac artery of the anaesthetized pig. *J. Physiol*. **573,** 133–145.
24. Florian, J.A., Kosky, J.R., Ainslie, K., Pang, Z., Dull, R.O., and Tarbell, J.M. (2003) Heparan sulfate proteoglycan is a mechanosensor on endothelial cells. *Circ. Res*. **93,** e136–142.
25. Thi, M.M., Tarbell, J.M., Weinbaum, S., and Spray, D.C. (2004) The role of the glycocalyx in reorganization of the actin cytoskeleton under fluid shear stress: a "bumper-car" model. *Proc. Natl. Acad. Sci. U. S. A.* **101,** 16483–16488.
26. Weinbaum, S., Zhang, X., Han, Y., Vink, H., and Cowin, S.C. (2003) Mechanotransduction and flow across the endothelial glycocalyx. *Proc. Natl. Acad. Sci. U. S. A.* **100,** 7988–7995.
27. Henry, C.B. and Duling, B.R. (1999) Permeation of the luminal capillary glycocalyx is determined by hyaluronan. *Am. J. Physiol*. **277,** H508–514.
28. Henry, C.B. and Duling, B.R. (2000) TNF-alpha increases entry of macromolecules into luminal endothelial cell glycocalyx. *Am. J. Physiol. Heart Circ. Physiol*. **279,** H2815–2823.
29. Squire, J.M., Chew, M., Nneji, G., Neal, C., Barry, J., and Michel, C. (2001) Quasi-periodic substructure in the microvessel endothelial glycocalyx: a possible explanation for molecular filtering? *J. Struct. Biol*. **136,** 239–255.
30. Vink, H. and Duling, B.R. (1996) Identification of distinct luminal domains for macromolecules, erythrocytes, and leukocytes within mammalian capillaries. *Circ. Res*. **79,** 581–589.
31. Chappell, D., Jacob, M., Hofmann-Kiefer, K., Conzen, P., and Rehm, M. (2008) A rational approach to perioperative fluid management. *Anesthesiology* **109,** 723–740.
32. Jacob, M., Chappell, D., and Rehm, M. (2009) The 'third space'--fact or fiction? *Best Pract. Res. Clin. Anaesthesiol.* **23,** 145–157.
33. Davies, P.F., Barbee, K.A., Volin, M.V., Robotewskyj, A., Chen, J., Joseph, L., Griem, M.L., Wernick, M.N., Jacobs, E., Polacek, D.C., et al. (1997) Spatial relationships in early signaling events of flow-mediated endothelial mechanotransduction. *Annu. Rev. Physiol*. **59,** 527–549.
34. Tarbell, J.M., Weinbaum, S., and Kamm, R.D. (2005) Cellular fluid mechanics and mechanotransduction. *Ann. Biomed. Eng*. **33,** 1719–1723.
35. Dudek, S.M. and Garcia, J.G. (2001) Cytoskeletal regulation of pulmonary vascular permeability. *J. Appl. Physiol.* **91,** 1487–1500.

36. Steppan, J., Hofer, S., Funke, B., Brenner, T., Henrich, M., Martin, E., Weitz, J., Hofmann, U., and Weigand, M.A. (2009) Sepsis and major abdominal surgery lead to flaking of the endothelial glycocalix. *J. Surg. Res*. [Epub ahead of print]
37. Nelson, A., Berkestedt, I., Schmidtchen, A., Ljunggren, L., and Bodelsson, M. (2008) Increased levels of glycosaminoglycans during septic shock: relation to mortality and the antibacterial actions of plasma. *Shock* **30,** 623–627.
38. Bernfield, M., Gotte, M., Park, P.W., Reizes, O., Fitzgerald, M.L., Lincecum, J., and Zako, M. (1999) Functions of cell surface heparan sulfate proteoglycans. *Annu. Rev. Biochem*. **68,** 729–777.
39. Li, J.P. and Vlodavsky, I. (2009) Heparin, heparan sulfate and heparanase in inflammatory reactions. *Thromb. Haemost*. **102,** 823–828.
40. Andriopoulou, P., Navarro, P., Zanetti, A., Lampugnani, M.G., and Dejana, E. (1999) Histamine induces tyrosine phosphorylation of endothelial cell-to-cell adherens junctions. *Arterioscler. Thromb. Vasc. Biol.* **19,** 2286–2297.
41. Aschner, J.L., Lum, H., Fletcher, P.W., and Malik, A.B. (1997) Bradykinin- and thrombin-induced increases in endothelial permeability occur independently of phospholipase C but require protein kinase C activation. *J. Cell Physiol.* **173,** 387–396.
42. Hippenstiel, S., Krull, M., Ikemann, A., Risau, W., Clauss, M., and Suttorp, N. (1998) VEGF induces hyperpermeability by a direct action on endothelial cells. *Am. J. Physiol.* **274,** L678–684.
43. Rotundo, R.F., Curtis, T.M., Shah, M.D., Gao, B., Mastrangelo, A., LaFlamme, S.E., and Saba, T.M. (2002) TNF-alpha disruption of lung endothelial integrity: reduced integrin mediated adhesion to fibronectin. *Am. J. Physiol. Lung Cell. Mol. Physiol.* **282,** L316–329.
44. Nieuwdorp, M., Mooij, H.L., Kroon, J., Atasever, B., Spaan, J.A., Ince, C., Holleman, F., Diamant, M., Heine, R.J., Hoekstra, J.B., et al. (2006a) Endothelial glycocalyx damage coincides with microalbuminuria in type 1 diabetes. *Diabetes* **55,** 1127–1132.
45. Nieuwdorp, M., van Haeften, T.W., Gouverneur, M.C., Mooij, H.L., van Lieshout, M.H., Levi, M., Meijers, J.C., Holleman, F., Hoekstra, J.B., Vink, H., et al. (2006b) Loss of endothelial glycocalyx during acute hyperglycemia coincides with endothelial dysfunction and coagulation activation in vivo. *Diabetes* **55,** 480–486.
46. Rehm, M., Bruegger, D., Christ, F., Conzen, P., Thiel, M., Jacob, M., Chappell, D., Stoeckelhuber, M., Welsch, U., Reichart, B., et al. (2007) Shedding of the endothelial glycocalyx in patients undergoing major vascular surgery with global and regional ischemia. *Circulation* **116,** 1896–1906.
47. Wang, L. and Dudek, S.M. (2009) Regulation of vascular permeability by sphingosine 1-phosphate. *Microvasc. Res.* **77,** 39–45.
48. Chappell, D., Hofmann-Kiefer, K., Jacob, M., Rehm, M., Briegel, J., Welsch, U., Conzen, P., and Becker, B.F. (2009) TNF-alpha induced shedding of the endothelial glycocalyx is prevented by hydrocortisone and antithrombin. *Basic Res. Cardiol.* **104,** 78–89.
49. Marechal, X., Favory, R., Joulin, O., Montaigne, D., Hassoun, S., Decoster, B., Zerimech, F., and Neviere, R. (2008) Endothelial glycocalyx damage during endotoxemia coincides with microcirculatory dysfunction and vascular oxidative stress. *Shock* **29,** 572–576.
50. Dull, R.O., Dinavahi, R., Schwartz, L., Humphries, D.E., Berry, D., Sasisekharan, R., and Garcia, J.G. (2003) Lung endothelial heparan sulfates mediate cationic peptide-induced barrier dysfunction: a new role for the glycocalyx. *Am. J. Physiol. Lung Cell. Mol. Physiol.* **285,** L986–995.
51. Zimmermann, P. and David, G. (1999) The syndecans, tuners of transmembrane signaling. *FASEB J.* **13(Suppl),** S91–S100.
52. Hayashida, K., Parks, W.C., and Park, P.W. (2009) Syndecan-1 shedding facilitates the resolution of neutrophilic inflammation by removing sequestered CXC chemokines. *Blood* **114,** 3033–3043.
53. Haynes, A., 3rd, Ruda, F., Oliver, J., Hamood, A.N., Griswold, J.A., Park, P.W., and Rumbaugh, K.P. (2005) Syndecan 1 shedding contributes to Pseudomonas aeruginosa sepsis. *Infect. Immun.* **73,** 7914–7921.
54. Hayashida, K., Chen, Y., Bartlett, A.H., and Park, P.W. (2008) Syndecan-1 is an in vivo suppressor of Gram-positive toxic shock. *J. Biol. Chem.* **283,** 19895–19903.
55. Marx, G. (2003) Fluid therapy in sepsis with capillary leakage. *Eur. J. Anaesthesiol.* **20,** 429–442.
56. Cheng, A.T., Plank, L.D., and Hill, G.L. (1998) Prolonged overexpansion of extracellular water in elderly patients with sepsis. *Arch. Surg.* **133,** 745–751.
57. Jacob, M., Rehm, M., Orth, V., Lotsch, M., Brechtelsbauer, H., Weninger, E., and Finsterer, U. (2003) Exact measurement of the volume effect of 6% hydoxyethyl starch 130/0.4 (Voluven) during acute preoperative normovolemic hemodilution. *Anaesthesist* **52,** 896–904.
58. Perko, M.J., Jarnvig, I.L., Hojgaard-Rasmussen, N., Eliasen, K., and Arendrup, H. (2001) Electric impedance for evaluation of body fluid balance in cardiac surgical patients. *J. Cardiothorac. Vasc. Anesth.* **15,** 44–48.
59. Rehm, M., Orth, V., Kreimeier, U., Thiel, M., Haller, M., Brechtelsbauer, H., and Finsterer, U. (2000) Changes in intravascular volume during acute normovolemic hemodilution and intraoperative retransfusion in patients with radical hysterectomy. *Anesthesiology* **92,** 657–664.
60. Rehm, M., Orth, V.H., Weninger, E., Jacob, M., Mayer, S., Brechtelsbauer, H., and Finsterer, U. (2001) Acute "normovolemic" hemodilution with 3.5% polygel (Haemaccel) for patients in the Wertheim-Meigs-operation. Blood loss of 87% blood volume without perioperative blood transfusion. *Anaesthesist* **50,** 580–584.

61. Weigand, M.A., Bardenheuer, H.J., and Bottiger, B.W. (2003) Clinical management of patients with sepsis. *Anaesthesist* **52,** 3–22.
62. Rehm, M., Haller, M., Orth, V., Kreimeier, U., Jacob, M., Dressel, H., Mayer, S., Brechtelsbauer, H., and Finsterer, U. (2001) Changes in blood volume and hematocrit during acute preoperative volume loading with 5% albumin or 6% hetastarch solutions in patients before radical hysterectomy. *Anesthesiology* **95,** 849–856.
63. Brunkhorst, F.M., Engel, C., Bloos, F., Meier-Hellmann, A., Ragaller, M., Weiler, N., Moerer, O., Gruendling, M., Oppert, M., Grond, S., et al. (2008) Intensive insulin therapy and pentastarch resuscitation in severe sepsis. *N. Engl. J. Med.* **358,** 125–139.
64. Rivers, E., Nguyen, B., Havstad, S., Ressler, J., Muzzin, A., Knoblich, B., Peterson, E., and Tomlanovich, M. (2001) Early goal-directed therapy in the treatment of severe sepsis and septic shock. *N. Engl. J. Med.* **345,** 1368–1377.
65. Connors, A.F., Jr., Speroff, T., Dawson, N.V., Thomas, C., Harrell, F.E., Jr., Wagner, D., Desbiens, N., Goldman, L., Wu, A.W., Califf, R.M., et al. (1996) The effectiveness of right heart catheterization in the initial care of critically ill patients. SUPPORT Investigators. *JAMA* **276,** 889–897.
66. Vincent, J.L. (2001) Hemodynamic support in septic shock. *Intensive Care Med*. **27(Suppl 1),** S80–92.
67. Wheeler, A.P. and Bernard, G.R. (1999) Treating patients with severe sepsis. *N. Engl. J. Med.* **340,** 207–214.

**This article should be cited as follows:**

Henrich, M., Gruss, M., and Weigand, M.A. (2010) Sepsis-induced degradation of endothelial glycocalix. *TheScientificWorldJOURNAL* **10**, 917–923. DOI 10.1100/tsw.2010.88.
